# Live imaging of human airway epithelial repair in precision-cut lung slices after targeted cell damage

**DOI:** 10.1016/j.bbrep.2026.102470

**Published:** 2026-01-29

**Authors:** Lara Gentemann, Fabian Röpken, Philipp Joel Mroch, Nils Noltemeyer, Sören Donath, Anna Elisabeth Seidler, Christopher Werlein, Patrick Zardo, Lavinia Neubert, Danny Jonigk, Hans-Gerd Fieguth, Alexander Heisterkamp, Katherina Sewald, Stefan Kalies

**Affiliations:** a Institute of Quantum Optics, Leibniz University Hannover, Hannover, Germany; b Lower Saxony Center for Biomedical Engineering, Implant Research and Development, Hannover, Germany; c REBIRTH Research Center for Translational Regenerative Medicine, Hannover, Germany; d Fraunhofer Institute for Toxicology and Experimental Medicine (ITEM), Hannover, Germany; e Biomedical Research in Endstage and Obstructive Lung Disease Hannover (BREATH), German Center for Lung Research (DZL), Hannover, Germany; f Institute of Pathology, Hannover Medical School, Hannover, Germany; g Department of Cardiothoracic Transplantation and Vascular Surgery, Hannover Medical School, Hannover, Germany; h Institute of Pathology, RWTH Aachen University Medical Faculty, Aachen, Germany; i KRH Clinics Hannover, Hannover, Germany

**Keywords:** Human precision-cut lung slices, Airway epithelial cells, Femtosecond laser nanosurgery, Precise damage induction, Localized injury repair, Lung damage

## Abstract

Precision-cut lung slices (PCLS) are a complex three-dimensional *ex vivo* model system comprised of all resident cell types of the lung, thus closely mimicking the *in vivo* situation in regards to structural composition and function. The herein described application of a precise airway epithelial lesion via femtosecond laser-based nanosurgery and subsequent longitudinal imaging via two-photon or confocal microscopy enables the examination of the tissue's repair responses on a single-cell level. Allowing for live observation of intercellular cross-talk, this study demonstrates an endogenous repair program is induced in human PCLS upon damage induction. As early reaction to a small epithelial lesion, physiological stress responses, including transient airway constriction and increased mucus secretion, occur, followed by epithelial restitution within 24 h. Automated cell detection and subsequent cell track analysis reveal a more linearly confined cellular movement in the course of repair. Further, non-stationary, motile cells directly interact with cell debris, thereby contributing to final resolution of the lesion. Together, these findings emphasize the suitability of PCLS, combined with localized laser-based damage induction and state-of-the-art microscopy techniques, as a model system to study complex intercellular interactions in the course of endogenous repair processes.

## Highlights

• Cellular repair processes in human PCLS can be identified via longitudinal imaging.
• Targeted cell ablation induces stress response and subsequent restitution.
• Non-stationary cells contribute to effective repair of small lesion in PCLS.

## Graphical abstract

**Figure:**
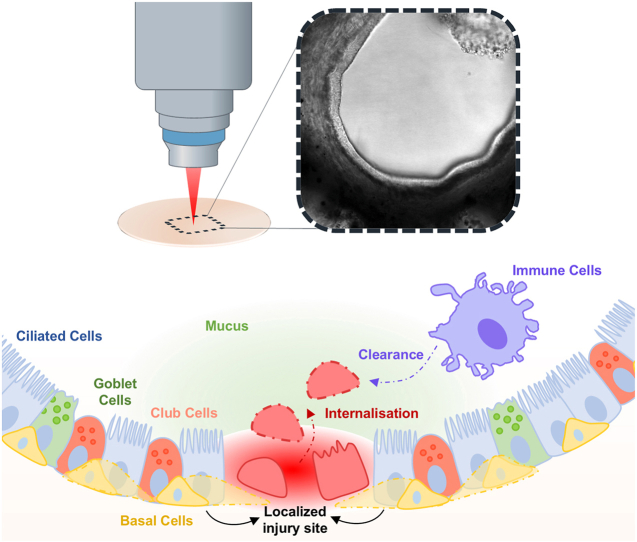

## 1 Introduction

Respiratory diseases are one of the world's leading causes of death [1]. Hence, many studies focus on investigating the mechanisms underlying abnormal lung repair in the context of various diseases [[2], [3], [4], [5], [6]]. Acute lung damage, often reliant on confined epithelial injury, is considered to lead to chronic conditions over an extended period of time, driven by accumulations of lesions that exceed the tissue's regenerative capabilities [7,8]. The initial localized microdamage can be caused by a multitude of stressors leading to potentially harmful immune responses [9,10]. The exposure to airborne toxic agents for instance can result in oxidative stress, activation of pro-inflammatory signaling pathways and DNA damage in epithelial cells [[11], [12], [13], [14]]. Similarly, viral or bacterial pathogens can target and damage respiratory epithelial cells, thereby inducing the release of cytokines and damage-associated molecular patterns (DAMPs), which in turn can trigger an immune response [15,16]. As a consequence of localized epithelial cell loss, the epithelial barrier's integrity is disturbed and an inflammatory cascade may be induced, driving a vicious circle finally resulting in further epithelial damage and potential remodeling processes, thereby decreasing the tissue's regenerative capacity over time [17,18]. Hence, localized injury in the proximal airway epithelium does not only induce, but also acts as modulator and enhancer of acute lung damage.

Tissue repair processes depend on a complex intercellular cross-talk [[19], [20], [21]]. In the proximal respiratory epithelium, especially basal cells, the tissue's resident stem cells, play an important role in restoring the epithelial integrity upon injury by self-renewal and as progenitors for secretory and ciliated cells [22]. Epithelial cells further interact with macrophages through secreted signals. Macrophages, as phagocytes, support clearing pathogens and cellular debris, while also regulating inflammation by secretion of cytokines [[23], [24], [25]]. Wound healing further relies on remodeling of the extracellular matrix, which is driven by fibroblasts [26,27]. For efficient repair, all these processes need to be strictly regulated, and can, if dysregulated, result in pathologic conditions such as fibrosis, or excessive and persistent inflammation. To study these processes, model systems closely resembling the native tissue's composition are needed. Numerous lung organotypic model systems mimicking the complexity of the entire organism to different degrees have been developed [22,[28], [29], [30], [31], [32]]. While there are multiple studies of co-culturing either airway epithelial-layers in air-liquid-interfaces (ALI) or organoid cultures with, for example, immunocytes, fibroblasts or mesenchymal cells, these systems still lack the broad cellular variety and the connective tissue structures present in native lung tissue and are additionally mostly based on pluripotent or adult stem cells [[33], [34], [35], [36], [37]]. Precision-cut lung slices (PCLS) serve as an *ex vivo* model that closely mimics the three-dimensional structural organization of the respiratory system. This approach preserves much of the functional integrity observed *in vivo*, enabling the study of lung physiology and pathology in a controlled setting [[38], [39], [40], [41], [42], [43], [44], [45]].

To gain a deeper understanding of endogenous reparative mechanisms, studying cellular responses to small lesions is of great interest. Conventional *in vitro* damage models, such as chemical or mechanical triggers, mostly lead to broad injury, which complicates the analysis of localized effects and cellular interactions *in situ*. In a recent approach, Bankole et al. implemented an acid injury and repair model to enable spatially restricted damage induction [46]. However, the injury site still comprises an entire region with a size defined by the diameter of a cylinder in which the chemical is instilled. In contrast, our group uses a femtosecond (fs) laser system to precisely manipulate single cells in complex cellular systems. This technique, termed fs laser nanosurgery can be applied to generate targeted and highly localized injury with the possibility to target subcellular structures, single or multiple cells [[47], [48], [49], [50], [51], [52]], thereby enabling the examination of the damaged cells' microenvironment's reaction and its reparative response primarily regulated by neighboring and immune cells, which is mechanistically distinct from repair processes in conventional damage models [49,[53], [54], [55]]. Fs laser nanosurgery has previously been employed to selectively remove subcellular structures in 2D cell culture, such as single z-discs in cardiomyocytes [49,50]. Recently, we analyzed the impact of single or multiple cell ablation within 3D epithelial mouse colon and airway organoids [51,52]. In airway organoids, we showed that a minor lesion caused by fs laser-based ablation of ten cells induced a reparative response recapitulating key aspects of *in vivo* epithelial restitution, driven by early cell migration and subsequent proliferation, finally leading to the restoration of the epithelial integrity within 24 h [52]. However, airway organoids missed the involvement of immune cells and other (mesenchymal) cells and connective tissue present in PCLS. A previous study of fs laser ablation in PCLS analyzed the immediate response of the tissue to injury, in comparison to a microneedle [56], reporting airway constriction accompanied by Ca^2+^ waves as an immediate tissue damage response. However, the study did not address the subsequent tissue repair or the intercellular interaction that may play a role in this. Similarly, continuous real-time imaging of PCLS was previously conducted to gain a deeper understanding of alveologenesis [42], lung immune cell localizations [57], and dynamic interactions of cells and the tissue's extracellular matrix [58], or to display the correlation between airway contraction and smooth muscle cell calcium signaling [59,60], but we did not find any reports about employing this approach of 4D live imaging for studies of acute repair processes.

In this study, we employed human PCLS (hPCLS) in combination with fs laser-based nanosurgery for localized airway epithelial cell ablation, and continuous two-photon or confocal microscopic imaging, followed by state-of-the-art image analysis pipelines, including automated cell detection and cell track analysis, to investigate reparative processes of the airway epithelium in a close-to-native setting, thereby providing establishing work for future use of this model in mechanistic repair studies.

## 2 Material and methods

### 2.1 Precision-cut lung slice generation, viability testing and cultivation

Primary human lung material was obtained from Hannover Medical School (MHH; Hannover) and Siloah Hospital (KRH; Hannover). All experiments with human lung tissue were approved by the Ethics Committee of Hannover Medical School and conducted in accordance with the Code of Ethics of the World Medical Association (renewed on April 22, 2015, number 2701-2015). All patients or their relatives, guardians, or custodians provided written informed consent for the use of lung tissue for research. All information regarding the identity of the patients was anonymized.

Tumor-free sections of tissue retrieved from patients undergoing tumor resections were used to generate PCLS, as described previously [61]. Briefly, lung lobes were filled with DMEM/F12 (Gibco) containing 2 % liquid, low-melting agarose (Fisher bioreagents). After polymerization on ice for 1 h, tissue was sliced into approximately 1 cm thick slices. Cylindrical cores with a diameter of 8 mm were stamped out and cut into 300–400 μm thin slices in Earl's Balanced Salt Solution using a Krumdiek tissue slicer (Alabama R&D). PCLS were washed three times with DMEM/F12 containing 100 U/mL penicillin and 100 μg/mL streptomycin, subsequently checked for the presence of airways, and transferred to a 48-well plate. One PCLS per well was cultured at 37 °C and 5 % CO_2_ in a humidified atmosphere. Only PCLS with intact airways were used for the experiments, which was determined by light microscopy based on the ciliary beat. To test the viability of each donor, LDH release and metabolic activity were analyzed using LDH assay (Roche) and Wst-1 assay (Roche), following the manufacturer's instructions.

### 2.2 Stainings

hPCLS were stained with various markers for live cell imaging according to the experiment's needs. In all hPCLS, nuclei were stained with Hoechst 33342 (Sigma-Aldrich, MO, United States, 1 μg/mL in hPCLS media) for 20 min at 37 °C, followed by washing with DPBS twice, prior to laser-mediated damage induction to enable fluorescent live cell imaging. For subsequent image acquisition using confocal microscopy, hPCLS were additionally incubated with NucSpot® Live 650 Nuclear Stain (Biotium, 1:500 in hPCLS media) for 45 min prior to imaging, and staining solution was kept on the probe during experimentation. Staining with wheat germ agglutinin (WGA) conjugated to Alexa Fluor 488 (Thermo Fisher, 10 μg/mL in hPCLS media, kept on during experimentation) was performed for fluorescent visualization of the airway's mucus layer. For visualization of live cells, hPCLS were stained using Calcein AM (Invitrogen, 2 mM in hPCLS media) for 30 min at 37 °C, followed by washing with DPBS twice. For live labeling of CD11b^+^ cells, immunostaining with PE-conjugated anti-CD11b antibody (clone M1/70, BioLegend (Cat# 101201, Lot# B411926)), 1:50 in hPCLS media +10 % FCS) was performed. hPCLS were incubated with antibody staining solution for 2 h at 37 °C, followed by three washing steps with hPCLS media prior to imaging in hPCLS media.

For proliferation analysis, a 5-Ethinyl-2′-Desoxyuridin-assay (EdU assay) as well as Ki67 (Mib1) immunostaining were conducted. For the EdU assay, hPCLS media was supplemented with 10 μM 5-EdU (Jena Bioscience, Germany) and hPCLS were incubated at 37 °C, 5 % CO_2_ for 2 h prior to subsequent laser-based cell ablation and live cell imaging. At time points from 6 h to 10 h post cell ablation, hPCLS were fixed by incubation with 2 % PFA (Carl Roth, Germany) in DPBS (Sigma-Aldrich, MO, United States) overnight at 4 °C. Cell permeabilization was conducted by incubation in 0.3 % Triton X-100 (Carl Roth, Germany) in DPBS for 2 h on a shaker at 130 rpm and RT. A subsequent click-chemistry-mediated staining reaction was achieved by incubation of hPCLS in the reaction mix (1 mM Cu2SO4 (Jena Bioscience, Germany), 10 mM sodium ascorbate (Jena Bioscience, Germany), 80 nM Cy5 azide (Jena Bioscience, Germany) diluted in DPBS (Sigma-Aldrich, MO, United States)) for 1 h at 37 °C in the dark, leading to fluorescent visualization of EdU that was incorporated into the DNA. For Ki67 immunostaining, hPCLS were fixed by incubation with 2 % PFA in DPBS overnight at 4 °C, followed by three washing steps with DPBS for 10 min each. Permeabilization was conducted using 0.3 % Triton X-100 for 2 h at room temperature, again followed by three washing steps with DPBS for 10 min each. Blocking was performed by incubation in ROTI®Block (Carl Roth, Germany) for 30 min at room temperature. Subsequently, primary antibody (rb-*anti*-Ki67, clone SP6, Acris (Cat# DRM004), 1:200 in ROTI®Block) was incubated overnight at 4 °C. The next day, six washing steps with DPBS were perfomed for 1 h each, followed by another blocking step for 30 min at room temperature and subsequent incubation with secondary antibody (dk-*anti*-rb-IgG-Alexa Fluor 647, clone Poly4064, BioLegend (Cat# 406414, Lot# B324032), 1:500 in ROTI®Block) at 4 °C overnight. The next day, six washing steps using DPBS for 1 h each were conducted, and stained hPCLS were imaged using laser scanning confocal microscopy.

### 2.3 Laser setup, image acquisition and manipulation

A Chameleon Ultra II laser system with a pulse length of 140 fs and a repetition rate of 80 MHz, previously described, was employed [50]. Cell ablation of approximately ten neighboring airway epithelial cells, identified via their localization lining an airway within the tissue slice, was conducted with a wavelength of 730 nm, a pulse energy of 0.9 nJ, a dissection speed of 10 μm/s, and a 20x objective (NA 0.8, Zeiss). As we assumed that natural airway damage would affect both basal and luminal cells, a random cell population of ten cells was chosen for manipulation. hPCLS stained with Hoechst 33342 and Calcein AM were visualized via two-photon microscopy, using a 20x objective (NA 0.8, Zeiss), at an excitation wavelength of 730 nm and emission was detected by a photomultiplier tube (Hamamatsu Photonics, Japan) using an emission filter at 460 ± 20 nm (Hoechst visualization) or at 510–560 nm (Calcein AM visualization). Visualization of fluorescent Calcein AM labeling via two-photon microscopy was generally possible for approximately 5–8 h, varying for each sample. Confocal laser scanning microscopy (Leica TSC SP5), using a 25x water immersion objective (NA 0.95, Leica), was applied for imaging of WGA-Alexa Fluor 488-, PE-*anti*-CD11b-immuno-, NucSpot® Live 650 Stain-, EdU-Cy5- and Ki67-immunostained hPCLS using excitation laser lines at 488 nm, 543 nm and 633 nm, as well as for capturing the transmission channel. For longitudinal live imaging, images (z-stacks in the range from ±30 μm to ± 65 μm from the ablation plane with a step size of 4 μm) were captured every 10–60 min from pre to a maximum of 13 h, and, if applicable, again at 24 h post laser-based cell ablation. Prolonged image acquisition intervals were chosen to reduce photobleaching over time. hPCLS were kept under culture conditions (37 °C, 5 % CO_2_) throughout the experimental procedure.

### 2.4 Data and image analysis

Image data were processed using Python 3.10, including Cellpose (version 2) [62,63], and custom scripts, as well as Fiji including TrackMate plugin [64,65].

For morphological analysis, airway luminal area was measured using Fiji's wand (tracing) tool (tolerance: 20, mode: legacy, smooth if thresholded: true) applied to maximum intensity z-projections of airway captures at each time point.

An initial evaluation of the magnitude of cellular or mucus movement within an airway over time was carried out via particle image velocimetry (PIV)-based analysis, for which a custom Python script using the OpenPIV package was applied. For this, time series of NucSpot® Live 650- or WGA-Alexa 488-stained PCLS image data was used as input. PIV analysis was conducted with an interrogation window size set to 16 px and an 8 px-overlap between consecutive windows. The resulting motion vectors were visualized as accumulated heatmaps, to display the spatial distribution of cell or mucus movement over time.

For analysis of proliferation and movement behavior of cells within hPCLS, a multi-step analysis pipeline, schematically presented in Fig. S1, was developed. First, automated cell detection was realized via applying a custom-trained Cellpose model based on the pre-trained nuclei model. Single plane images of Hoechst-stained hPCLS were used for training (learning_rate: 0.01; weight_decays: 0.0001; n_epochs: 500). The final model was applied to all Hoechst-channel two-photon microscopy image data (flow_threshold: 2; cellprob_threshold: −1.5), and nuclei label images were generated for downstream analysis. Hyperstacks of nuclei label images (z-/t-dimensions resemble original captures) were further processed using either a custom Python script or Fiji Trackmate plugin. The Python script was applied to determine cell counts within the entirely captured field of view of each hPCLS, or within certain distances from the ablation site at time points from 0 to 10 h post laser-based damage induction. In this context, three distance bins of 100 μm, 200 μm and 300 μm were defined, counting all labeled cells in distances between 0–100 μm, 100–200 μm and 200–300 μm from the ablation site, respectively. For downstream analysis via Fiji Trackmate, cell detection was achieved via LoG Detector on label images (estimated object diameter: 8.2 μm; quality threshold: 8.42), and cell tracking via LAP Tracker (frame to frame linking: max distance: 24.6 μm; track segment gap closing: max distance: 82 μm, max frame gap: 4; track segment splitting or merging not allowed). The output spots files were saved and further processed using custom Python scripts for calculation of each individual cell's track features regarding its displacement, maximum distance traveled, total distance traveled, mean speed, mean straight line speed, mean directional change, confinement ratio, and linearity of forward progression. Definitions of track feature parameters and respective calculations are illustrated in Fig. S1B.

### 2.5 Quantification and statistical analysis

Statistical testing of cell count and movement analysis was performed by Mann-Whitney-U-test using Python with a statistical threshold of p < 0.05 (∗). Figures were created using Fiji, Python, Microsoft Powerpoint and Inkscape.

## 3 Results

### 3.1 Small epithelial lesions induce physiological stress reaction and damage repair

To investigate the impact of localized epithelial lesions, femtosecond (fs) laser-based cell ablation was applied to selectively target airway epithelial cells. The primary aim was to determine how such confined injury influences overall airway architecture, and to assess whether indicators of physiological stress and epithelial repair could be detected in a complex, human-based *ex vivo* culture system of PCLS. This approach was further used to evaluate the suitability of the method for studying injury-repair dynamics *in situ*.

**Fig. 1:**
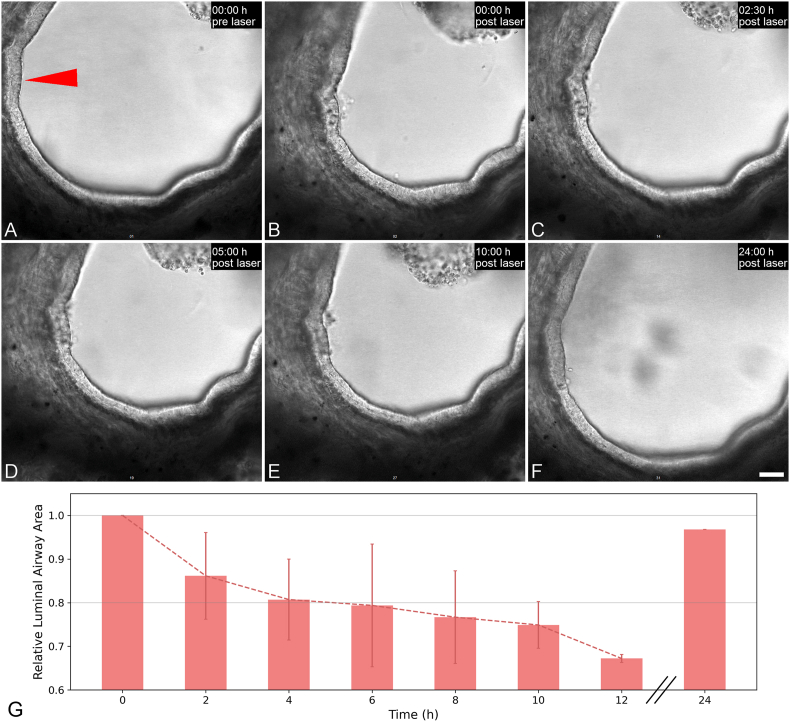
hPCLS morphology is restored upon targeted cell ablation. (A–F): Representative time series of single-plane transmission microscopy images of hPCLS at time points pre (A) and 0 h (B), 2.5 h (C), 5 h (D), 10 h (E) and 24 h (F) post laser-based cell ablation; red arrow: site of cell ablation. Scale bar: 50 μm. (G): Relative luminal airway area of hPCLS right before (0 h) and until 24 h post laser-based cell ablation. Data represents mean ± SD of n = 2 biological replicates.

**Targeted epithelial injury induces transient airway contraction:** In all examined airways, the epithelium formed a continuous lining of the luminal surface and displayed consistent ciliary beating prior to damage induction. A representative time series of confocal microscopy transmission channel images of an airway subjected to laser-mediated injury induction is shown in Fig. 1. It displays the airway before injury and the subsequent morphological changes up to 24 h post-ablation. Directly after laser-based cell ablation, the damaged site was marked by dark spots within the epithelial layer. Cells within the targeted area exhibited either partial or complete detachment from the surrounding epithelium and were frequently observed free-floating in the culture medium. Detached cells displayed a rounded morphology, consistent with the loss of their normal elongated profile.

Concomitant with these cellular alterations, the entire airway underwent a measurable contraction, with the luminal cross-sectional area decreasing to 70 ± 5 % of its initial size (Fig. 1B). While the apparent morphology of the epithelial damage site as well as the constricted airway lumen did not undergo notable changes until 10 h post cell ablation (Fig. 1C–E), with relative luminal airway area ranging from 86 ± 10 % (at 2 h) to 75 ± 5 % (at 10 h) from its initial size (Fig. 1G), it can be observed that the microscopically assessed epithelial structure and the airway's initial size were restored at 24 h post damage induction (Fig. 1F and G). This quantitative data is based on n = 2 observations only though, as other studied airways were bigger in size and did not allow a sufficiently broad capture of the airway via microscopic analysis to analyze the cross-sectional area.

**Fig. 2:**
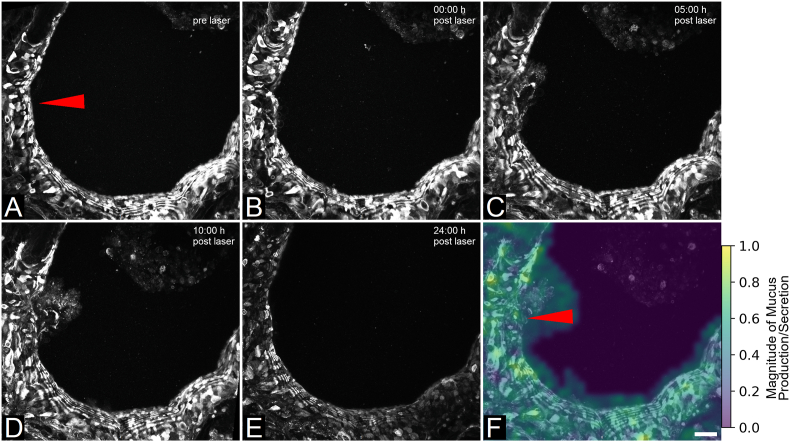
hPCLS mucus layer restitutes upon targeted cell ablation. (A–E): Representative time series of wheat germ agglutinin (WGA)-Alexa Fluor 488-stained hPCLS confocal microscopy images (max. intensity z-projection of captures ±65 μm of ablation site) at pre (A), 0 h (B), 5 h (C), 10 h (D) and 24 h (E) post laser-based ablation of epithelial cells. Red arrow: site of cell ablation. (F): Particle image velocimetry-based analysis of mucus distribution accumulated over 24 h. Data shows representative results of n = 3 biological replicates. WGA: mucus stain. Scale bar: 50 μm.

**Localized epithelial injury induced elevated mucus secretion at damage site:** As a physiological stress response, airway epithelial cells are described to produce and secrete an elevated amount of mucus [66,67]. Hence, hPCLS stained with Alexa Fluor 488-conjugated wheat germ agglutinin (WGA) enabled live-visualization of mucus secretion dynamics (Fig. 2, Video S1). As expected, a disruption of the even mucus layer lining the airway's apical surface was observed already shortly after laser-mediated cell ablation (Fig. 2A and B). Over the course of 10 h following injury induction, epithelial cells surrounding the damaged area secreted an increasing amount of mucus (Fig. 2B–D). At 24 h post laser manipulation, the excess mucus had been cleared from the airway and a restitution of the initially even mucus layer could be observed (Fig. 2E). The magnitude of mucus production and secretion within the represented airway over time was further visualized as result of a particle image velocimetry-based image analysis. Applying this method, pixel-based alterations over time within the fluorescence image data are displayed in a heatmap-based image, showing the magnitude of mucus motion, which underlines the high mucus production by cells in close proximity to the damage site and the luminal mucus overload especially around this area (Fig. 2F).

**Fig. 3:**
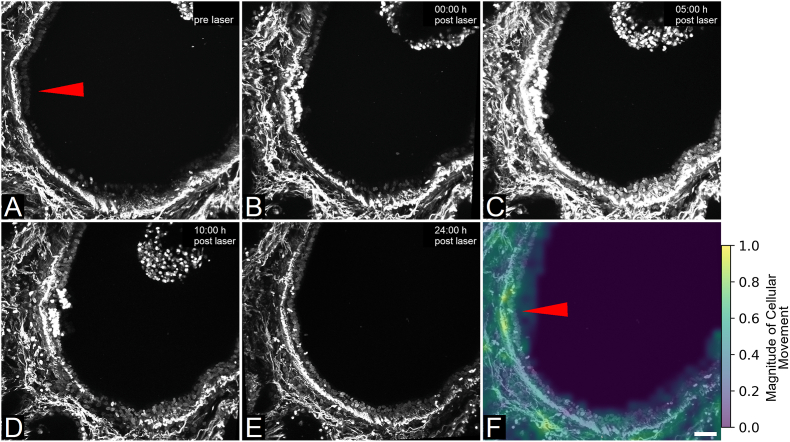
hPCLS epithelial repair involves cellular movement. (A–E): Representative time series of NucSpot® Live 650-stained hPCLS confocal microscopy images (max. intensity z-projection of captures ±25 μm of ablation site) at pre (A), 0 h (B), 5 h (C), 10 h (D) and 24 h (E) post laser-based ablation of epithelial cells. Red arrow: site of cell ablation, NucSpot® Live 650: nuclear stain. (F): Particle image velocimetry-based analysis of cellular movement accumulated over 24 h. Data shows representative results of n = 5 biological replicates. Scale bar: 50 μm.

**Repair of small lesions involves cell shedding and increased cellular movement in neighboring area:** Confocal microscopy images of hPCLS stained with NucSpot® Live 650 for nuclei visualization allowed for further evaluation of airway epithelial repair, thereby supporting observations made based on morphology (Fig. 3, **Video S2**). While the tissue displayed strong autofluorescence originating from various connective tissue structures when imaging NucSpot® Live 650, nuclei were clearly identifiable. It can be observed that damaged cells, which showed a bright fluorescent signal upon laser-based manipulation, were slowly detached from the epithelial layer and invaginated into the airway's lumen (Fig. 3A–D). While this process had not been completed at 10 h after damage induction, the epithelial layer was restored at 24 h post laser manipulation. At this time point, the airway showed no signs of injury and no damaged cells or cell debris were observable within the field of view (Fig. 3E), indicating appropriate clearance had taken place in the meantime. A higher magnitude of movement around the damage area in comparison to other regions of the airway was indicated by a particle image velocimetry-based image analysis of the NucSpot® Live 650-stained cells (Fig. 3F). This suggests either proliferation or migration, or both processes, were stimulated in hPCLS airways upon targeted cell ablation.

### 3.2 Small lesion repair is mediated by cell movement rather than proliferation

Applying a more complex image analysis pipeline, captures of hPCLS with nuclear staining were used for cell count analysis. To ensure to conduct the analysis as unbiased as possible, we aimed for automated cell nuclei detection and tracking over time. Strong autofluorescence signals originating from connective tissue and extracellular matrix components in all captured images complicated cell detection though. Thus, we made use of Cellpose, a deep learning algorithm especially developed for cell and nucleus segmentation [62,63], with which a sufficient nuclei detection and a generation of label images for downstream processing was achieved.

**Fig. 4:**
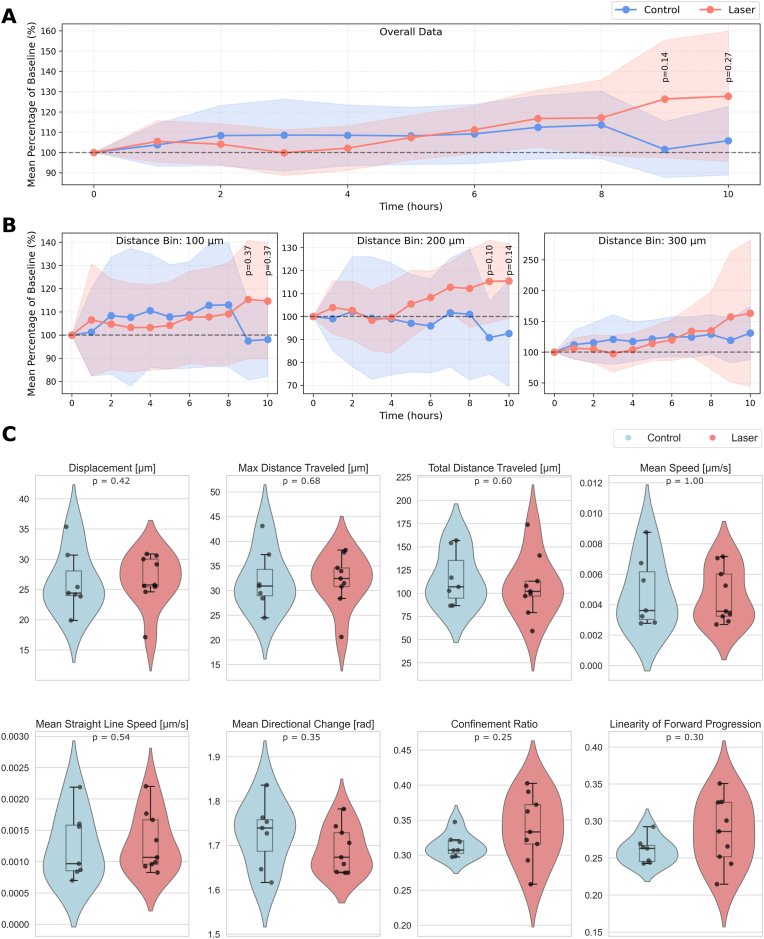
Cell count and movement analysis. (A,B): Cell counts indicate cell movement towards ablation site. Changes in cell count within PCLS airway epithelium over the course of time (0–10 h post ablation) within the entire field of view (A) or within distinct distances from the ablation site (B; distance bins: 100 μm (left), 200 μm (center) and 300 μm (right)). Data points of overall data refer to means of n = 7 (control) or n = 9 (laser-based ablation) experiments, colored area around shown mean values represent 95 % confidence intervals. (C): Cell track features indicate a higher grade of directed cellular movement in PCLS subjected to targeted cell ablation compared to untreated control. Each data point represents mean of all cell tracks detected per PCLS/experiment of n = 7 (control) or n = 9 (laser-based ablation) experiments. (A–C): Statistical testing was performed via Mann-Whitney-U test with ∗p < 0.05; non-significant unless stated otherwise.

**Localized epithelial injury leads to an elevated cell count near the damage site**: A custom Python script determined the number of cells within distinct distances from the ablation site of each airway based on the nuclei label images over the course of 10 h post laser manipulation (Fig. 4A and B). In control hPCLS, the cell count stayed nearly constant over the duration of the experiment, with only small fluctuations reaching a maximum of 114 ± 9 % of cells at 8 h compared to the initially determined baseline (p = 0.57 compared to baseline). Similarly, the cell count in hPCLS with lesions showed no significant alterations over the entire period observed. Still, at 9–10 h post damage induction, relative cell numbers tended to increase compared to control, reaching relative counts of 126 ± 15 % (p = 0.14 compared to control) and 128 ± 16 % (p = 0.27 compared to control) of its baseline. This tendency was also observed in distinct distance bins to the ablation site, as shown in Fig. 4B. While no statistically significant effects were observed, all three defined regions, within 100 μm (“close”), between 100 μm and 200 μm (“median”), and between 200 μm and 300 μm (“distant”) from the ablation site, were characterized by a slight increase in cell counts with progressing time in case of preceding laser manipulation. Especially in areas in “close” and “median” proximity to the ablation site, relative cell counts varied between treatment and control groups at 10 h post cell ablation, amounting to 115 ± 13 % (laser) vs. 98 ± 8 % (control) (close, p = 0.37) and 115.5 ± 8.3 % (laser) vs. 92.6 ± 11.8 % (control) (median, p = 0.14). In summary, for both overall as well as binned data, a trend of increased cell counts upon damage induction compared to control was observed, though not statistically significant.

**Proliferation is not enhanced upon confined epithelial damage induction**: As both proliferation and migration might have attributed to varying cell counts, cell proliferation was investigated via EdU assay and Ki67 immunostaining. These endpoint analysis approaches were conducted at various time points (6–10 h post laser-mediated cell ablation) and showed an overall vanishingly low number of proliferating cells within the airway epithelium, both in control PCLS as well as in case of damage induction. Generally, these few proliferating cells were distributed rather randomly over the entire airway, and no clusters of proliferative cells, potentially indicating a specifically induced proliferation, were detected. These results indicate that early epithelial repair following laser-based damage induction did not rely on enhanced proliferation activity.

**Cell track feature patterns indicate epithelial injury enhanced directed cell movement:** For a more detailed analysis of cell movement within the observed regions in hPCLS, Cellpose-generated nuclei label images were further processed using Fiji Trackmate for automated detection of individual cell tracks over time, followed by a final analysis of data with regards to various track features using a custom Python script. It needs to be noted that only cell tracks from images capturing the first 10 h post lesion were analyzed, therefore results display the epithelium's initial reparative response only. Though not statistically significant, tendencies of altered track feature patterns in hPCLS subjected to laser-induced lesions compared to control could be observed (Fig. 4C). Looking at the displacement, which describes the distance between a cell's start and end point, median values of cells within hPCLS subjected to laser-based cell ablation and untreated control (27 ± 1 μm and 26 ± 2 μm, p = 0.41) were nearly the same. Still, the violin plot showed an altered distribution with an enhanced density at slightly higher displacement values in case of laser treatment compared to control. A similar pattern was observed for the maximum distance traveled (p = 0.68), which described the distance between the farthest apart locations a cell encountered. For the total distance traveled, which summarizes the distances of the entire cell's track, a bimodal distribution with a second enhanced density at higher values was detected in case of control hPCLS. A comparable bimodal distribution of data points could also be observed for the cells' mean speed upon laser treatment, but not in control hPCLS. Beside these measured sizes, four parameters, indicating the efficacy of a cellular movement, were calculated (Fig. 4C bottom row): In case of mean straight line speed, both median values as well as overall density distribution did not show any differences between conditions. The mean directional change, which takes the angular changes of a cell's path into account, showed a shifted density plot distribution upon laser manipulation (p = 0.35). The violin plots of both confinement ratio, a measure of how efficient a cell's displacement was, and linearity of forward progression showed a distinctly altered shape for laser-treated hPCLS compared to control. While the density distribution of both parameters covered a wider range in hPCLS subjected to lesions than in control, the median values were elevated in both cases, resulting in 0.34 ± 0.02 (laser) versus 0.31 ± 0.01 (control) for confinement ratio (p = 0.25), and 0.28 ± 0.01 (laser) versus 0.26 ± 0.01 (control) for linearity of forward progression (p = 0.30). Together, these track feature patterns indicated a higher grade of directed cell movement within hPCLS airways previously subjected to laser-based cell ablation compared to control. These findings further support the cell count analysis results, which showed a trend of increasing cell counts in areas around the ablation site.

### 3.3 Non-stationary cells involvement in airway epithelial lesion repair in hPCLS

hPCLS preserve the native tissue's cellular composition, thereby providing an *ex vivo* environment that retains resident and circulating cell populations. In combination with high spatio-temporal imaging, this allows not only the analysis of cell movement dynamics within the epithelial cell population but also the investigation of intercellular interactions and their roles in epithelial repair processes following targeted localized injury induction.

**Fig. 5:**
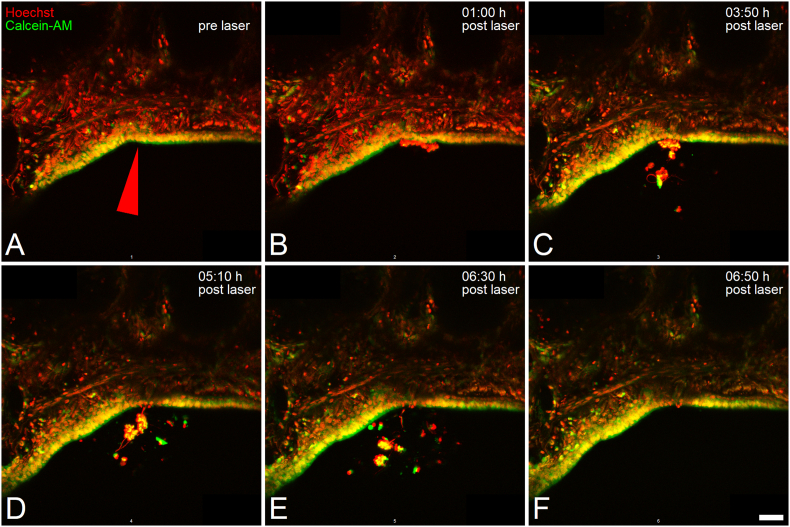
Recruitment of presumably immune cells to damage site. (A–F): Exemplary time series of Hoechst- (red) and Calcein-AM-stained (green) hPCLS multiphoton microscopy images (max. intensity z-projection of captures ±25 μm of ablation site) at pre (A), as well as 1 h (B), 3:50 h (C), 5:10 h (D), 6:30 h (E) and 6:50 h (F) post laser-based ablation of epithelial cells. Data shown is representative for observations made in n = 2 biological replicates. Scale bar: 50 μm.

**Potential immune cells mediate clearance of cell debris from damage site**: In this context, a contribution of non-stationary cells, presumably immune cells, in clearing dead cells/cell debris from the damage site was observed, representatively shown in Fig. 5 and **Video S3**. A similar behavior of non-stationary cells was not observed in case of control airways in which no injury was set (**Video S4**). For laser-based cell ablation, live cells within the epithelial layer, characterized by positive calcein-AM staining, were targeted (Fig. 5A). Within 1 h post damage induction, shedding of the dead cells into the airway's lumen took place (Fig. 5B). At 3:30 h upon laser treatment, previously non-present, calcein-AM-positive, cells firstly appeared within the airway's lumen in the captured field of view. Within the next 3 h, an apparently directed process was observed. This included a movement of these migrated cells towards the dead cell mass located on top of the epithelium's apical side (Fig. 5C), an enclosing and subsequent detachment of such from the epithelial layer, finally resulting in the removal of the cell debris from the damage site (Fig. 5D–F). It needs to be noted that final clearance of the dead cell mass from the captured field of view occurred rather abruptly within the time frame of 6:30 h and 6:50 h post cell ablation (Fig. 5E and F, Video S3), potentially due to an ultimate detachment of the cell debris from the ablation site and a subsequently accelerated clearance, possibly supported by passive cell floating. Though suggested by their characteristic behavior, the non-stationary cells in these captures, only stained with Hoechst and calcein-AM and lacking specific labeling, cannot explicitly be identified as immune cells.

Live-immunostaining of the immunocyte surface marker CD11b, characteristically expressed by, e.g., macrophages and dendritic cells [68], was therefore conducted prior to cell ablation and longitudinal imaging, indicating a potential involvement of CD11b^+^ cells in debris clearance (Fig. S2 and **Video S5**). As this cell behavior could only be observed in n = 1 studied hPCLS, further experimentation is needed for reliable interpretation though.

## 4 Discussion

Despite the airway epithelium's remarkable cellular plasticity and high reparative capacities, lung diseases originating in the (small) airways still pose one of the world's leading causes of death [1]. To better understand how pathologic conditions emerge and might be prevented, it is essential to also gain a deeper understanding of the early phases of tissue repair. By applying localized airway epithelial injury to hPCLS via fs laser-based cell ablation and subsequent continuous observation of the damage site, we demonstrate that processes induced by endogenous repair programs can be studied in a close-to-native environment with high spatio-temporal resolution. We show that the *ex vivo* model system reacts to damage induction with a physiological stress response, tendentially altered cell movement patterns and involvement of motile, potentially immune, cells to restore its airway epithelial integrity, marking it as suitable for application of future mechanistic repair studies.

We observed early physiological stress responses upon laser-based cell ablation, namely a reversible airway constriction and enhanced mucus secretion. Both processes have been described to occur as effects of, for example, mechanical or damage stimuli, in literature [8,56,67,69,70]. Having employed a similar model system as used in this study, Zhou et al. showed that in rat PCLS, a rapid global airway constriction to 70 % of its initial size was triggered upon laser-based ablation of a single airway epithelial cell [56], which is consistent with our observations. The contractions were found to be attributed to soluble mediators, especially adenosine-5′-triphosphate (ATP), released by the damaged epithelium, which subsequently evoked calcium oscillations in smooth muscle cells [56,71]. Other studies showed that nucleotides such as ATP mediate an enhanced mucus production and secretion by airway epithelial cells [69,[72], [73], [74], [75]]. On this basis, it can be suggested that the local increase in mucus secretion by cells in close proximity to the damage site observed here is mediated by paracrine effects of ATP release by the injured epithelium as well, thus representing a physiological stress reaction.

For epithelial restitution, a clearance of the dead cells is inevitable. In good agreement with our observations, various studies showed that, as a first step, dying or dead cells were actively extruded from the epithelial layer into the airway's lumen [52,76,77]. For closure of the resulting gap within the epithelial barrier, basal cell spreading, migration and subsequent proliferation are key steps in repair [[78], [79], [80], [81], [82]]. While we did not specifically investigate cell spreading, migration or golgi (re-)orientation, which can serve as indication for directed migration [83,84], the analyzed cell count and track features indicated a higher grade of linearly confined cell movement within the first 10 h upon injury in comparison to control samples. Combined with our results obtained regarding cell proliferation, which did not show any damage-induced changes in PCLS, and the observation of a morphologically restored epithelium within 24 h post damage induction, we presume the injury caused by targeted cell ablation was small enough to be initially restored by solely cell spreading and migration. Such a repair mechanism lacking the early involvement of cell proliferation was previously attributed to the fact that migration can occur notably faster and therefore poses a more secure process for rapid epithelial restitution [85,86]. We cannot exclude that proliferation would have been found to be upregulated at later time points and after the epithelial gap had been physically closed already though, as shown in a study by Gindele et al. [81] Besides an efficient wound closure, the complete tissue repair process further relies on a resolution of the dead cells, which often involves an immune cell contribution. In this context, it is notable that respiratory epithelial cells, as well as immune cells, express pattern recognition receptors, which facilitate sensing of not only pathogen-associated molecular patterns, but also damage-associated molecular patterns (DAMPs) passively released from the damaged cells that were shed into the airway's lumen [8,16,87]. Recognition of these DAMPs is known to cause a sterile inflammatory response by, on the one hand, directly activating immune cells in close proximity, and, on the other hand, inducing an intracellular signaling cascade within the epithelial cells finally leading to secretion of a wide range of cytokines and chemokines, subsequently attracting cells of the innate immune system [8,16,80,[87], [88], [89], [90], [91]]. In this context, Xie et al. demonstrated that ALI-cultured primary human airway epithelial cells react to mechanical damage with an upregulated expression of various chemokines responsible for immune cell recruitment, as, for example, CCL5, CCL2, CXCL2 and CXCL10, within a few hours [80]. On this basis, we presume, our observations of non-stationary cells seemingly being attracted to the damage site and removing the cell debris present injury-stimulated pro-repair innate immune responses which rely on preceding DAMP recognition and/or epithelial chemokine release. This assumption is supported by previous studies which showed the positive contribution of different immunocytes, such as airway macrophages, dendritic cells and neutrophils, on debris clearance and epithelial repair processes [57,[91], [92], [93], [94], [95], [96], [97]]. In this context, using an ALI co-culture model of LLC cells and macrophages, Ding et al. demonstrated sufficient transepithelial migration of macrophages upon bacterial challenge and subsequent phagocytic behavior to resolve infection [33]. Similarly, van Riet et al. employed an ALI co-culture model of primary human bronchiolar epithelial cells and monocyte-derived macrophages, in which the macrophages were observed to support the epithelial repair upon injury induction [34]. Airway macrophages are further described to play important roles in supporting tissue repair via efferocytosis of dying cells, and concomitant release of soluble mediators, thereby affecting structural cells and favoring, for example, migration [98]. Neutrophils were as well described to support the airway's debris clearance. Their entry into the luminal space via transepithelial migration often precedes a longer journey as they mostly originate from the circulatory system [[99], [100], [101]]. Based on this, it needs to be taken into consideration that the *ex vivo* model system of PCLS only comprises cell types resident within the tissue at the time of preparation. Concomitantly, it lacks a functional circulatory system, thus not allowing investigations of the involvement of immunocyte recruitment from the blood, for which an appropriate co-culture system would be needed, as, for example, established by Chang et al. [93] While the variety of blood-derived immune cells especially play important roles upon bacterial or viral challenge and associated epithelial injury [102,103], we cannot exclude their involvement in a localized damage repair investigated in this study without a respective model system. This in mind, we could assume the observed non-structural cells to be tissue-residing macrophages. Still, at the moment, this only poses a speculation on the basis of the cells' phenotypic behavior, but future live immunostainings utilizing a broader set of characteristic immune cell markers is needed to clarify which exact cell types are involved in the clearance of the cell debris upon injury in PCLS. Also, it is notable that such motile, potentially immune, cells were observed to contribute to debris clearance in only a few, but not all studied hPCLS, indicating that efficient epithelial repair might rely on varying processes to different extents. Further, it also needs to be taken into account that, *in vivo*, the mucociliary clearance along the respiratory tract poses an effective mechanism for the removal of cell debris. In contrast, the *ex vivo* model system PCLS lacks such an efficient mucociliary clearance mechanism, thereby potentially favoring immune cell involvement for efficient repair which might not be required and triggered in the *in vivo* situation. It is additionally noteworthy that the tissue's origin from human tumor resection material, which is common practice in the field, might pose a limitation for studying endogenous repair. As PCLS were generated from non-tumorous tissue sections, the tissue was generally assumed to be healthy. Still, in contrast to biological material from animals kept under strictly defined conditions, human donor material is characterized by large heterogeneity in between samples. This is due to the donors' potential comorbidities, preceding systemic (anti-tumor) treatment and medication, or general individual lifestyle, which we are blinded against. All these factors might affect the tissue's intrinsic functions in the context of repair, potentially impacting, e.g., the immune cell number and/or activity. While, for this reason, many studies still rely on animal models, the limits of transferability of such experiments to the human system need to be considered, as especially rodent lungs differ from humans' in regard to anatomy, cell composition and hence physiology [104,105]. Complying with the 3R principles [106], this underlines the importance of the development of appropriate human model systems for examination of airway epithelial repair. While, as previously discussed, the heterogeneity in human PCLS might pose a limitation, it at the same time reflects the naturally occurring heterogeneity in the human population and opens up opportunities for capturing cellular processes in a translationally relevant setting.

### 4.1 Conclusion

In conclusion, using a complex damage-repair model employing hPCLS, fs laser based nanosurgery, 4D imaging and sophisticated analysis pipelines, here, we demonstrated the feasibility of studying local airway epithelial repair processes in the context of a close-to-native tissue environment. By showing that hPCLS react to targeted injury induction with previously described behavior characteristic for the *in vivo* situation, such as airway constriction, enhanced mucus production, altered cell movement pattern and potential immunocyte contribution in damage repair, we demonstrated the functional and direct interactions of residing cells of different compartments, underlining the broad cellular functionality within the complex 3D *ex vivo* model system of hPCLS and the major advantage state-of-the art live microscopy implies.

Together, the findings presented here strikingly mark hPCLS as a suitable model to study repair processes on a single-cell level, e.g. individual cells’ motile behavior or intercellular interactions, when combined with targeted laser-based damage induction and longitudinal imaging approaches. The shown cross-talk between cells of different compartments emphasize that hPCLS react in a manner similar to the *in vivo* lung tissue. Hence, our results pave the way not only for further mechanistic investigation of physiologic repair, but also to study early processes driving abnormal repair by employing hPCLS derived from diseased patients.

## 5 Resource availability

### 5.1 Lead contact

Requests for further information and resources should be directed to and will be fulfilled by the lead contact, Stefan Kalies (kalies@iqo.uni-hannover.de).

### 5.2 Materials availability

This study did not generate new unique reagents.

### 5.3 Data and code availability

• All data reported in this data will be shared by the lead contact upon request.
• This paper does not report original code.
• Any additional information required to reanalyze the data reported in this paper is available from the lead contact upon request.

## CRediT authorship contribution statement

**Lara Gentemann:** Conceptualization, Data curation, Formal analysis, Investigation, Methodology, Software, Validation, Visualization, Writing – original draft, Writing – review & editing. **Fabian Röpken:** Methodology, Resources, Writing – review & editing. **Philipp Joel Mroch:** Methodology, Resources, Writing – review & editing. **Nils Noltemeyer:** Formal analysis, Software, Writing – review & editing. **Sören Donath:** Investigation, Writing – review & editing. **Anna Elisabeth Seidler:** Methodology, Visualization, Writing – review & editing. **Christopher Werlein:** Methodology, Resources, Writing – review & editing. **Patrick Zardo:** Methodology, Resources, Writing – review & editing. **Lavinia Neubert:** Methodology, Resources, Writing – review & editing. **Danny Jonigk:** Methodology, Resources, Writing – review & editing. **Hans-Gerd Fieguth:** Methodology, Resources, Writing – review & editing. **Alexander Heisterkamp:** Funding acquisition, Project administration, Supervision, Writing – review & editing. **Katherina Sewald:** Funding acquisition, Methodology, Project administration, Resources, Supervision, Writing – review & editing. **Stefan Kalies:** Conceptualization, Data curation, Formal analysis, Funding acquisition, Methodology, Project administration, Resources, Software, Supervision, Validation, Visualization, Writing – review & editing.

## Funding

The study was funded by the biomedical research in endstage and obstructive lung disease Hannover (BREATH) from the German Lung Center (DZL).

## Declaration of competing interest

The authors declare that they have no known competing financial interests or personal relationships that could have appeared to influence the work reported in this paper.

## Data Availability

Data will be made available on request.

## Appendix A Supplementary data

Supplementary Material Supplementary Material

VideoS1 VideoS1

VideoS2 VideoS2

VideoS3 VideoS3

VideoS4 VideoS4

VideoS5 VideoS5

## References

[1] Agusti A., Vogelmeier C.F., Halpin D.M.G. (2022). Tackling the global burden of lung disease through prevention and early diagnosis. Lancet Respir. Med..

[2] Andersson C.K., Iwasaki J., Cook J., Robinson P., Nagakumar P., Mogren S., Fleming L., Bush A., Saglani S., Lloyd C.M. (2020). Impaired airway epithelial cell wound‐healing capacity is associated with airway remodelling following RSV infection in severe preschool wheeze. Allergy.

[3] Pineau F., Shumyatsky G., Owuor N., Nalamala N., Kotnala S., Bolla S., Marchetti N., Kelsen S., Criner G.J., Sajjan U.S. (2020). Microarray analysis identifies defects in regenerative and immune response pathways in COPD airway basal cells. ERJ Open Res..

[4] Ghosh M., Miller Y.E., Nakachi I., Kwon J.B., Barón A.E., Brantley A.E., Merrick D.T., Franklin W.A., Keith R.L., Vandivier R.W. (2018). Exhaustion of airway basal progenitor cells in early and established chronic obstructive pulmonary disease. Am. J. Respir. Crit. Care Med..

[5] Gohy S.T., Hupin C., Fregimilicka C., Detry B.R., Bouzin C., Chevronay H.G., Lecocq M., Weynand B., Ladjemi M.Z., Pierreux C.E. (2015). Imprinting of the COPD airway epithelium for dedifferentiation and mesenchymal transition. Eur. Respir. J..

[6] Michael S., Liotta N., Fei T., Bendall M.L., Nixon D.F., Dopkins N. (2024). Endogenous retroelement expression in modeled airway epithelial repair. Microb. Infect..

[7] Mei Q., Liu Z., Zuo H., Yang Z., Qu J. (2022). Idiopathic pulmonary fibrosis: an update on pathogenesis. Front. Pharmacol..

[8] Raby K.L., Michaeloudes C., Tonkin J., Chung K.F., Bhavsar P.K. (2023). Mechanisms of airway epithelial injury and abnormal repair in asthma and COPD. Front. Immunol..

[9] Nash A.A., Dalziel R.G., Fitzgerald J.R. (2015). Mims' Pathogenesis of Infectious Disease.

[10] Cui C., Yang R., Chen H., Li D., Sun X., Wang Y., Pan Q. (2025). Air toxins disorder the NF-kB pathway leads to immune disorders and immune diseases in the human health. Ecotoxicol. Environ. Saf..

[11] Cipollina C., Bruno A., Fasola S., Cristaldi M., Patella B., Inguanta R., Vilasi A., Aiello G., La Grutta S., Torino C. (2022). Cellular and molecular signatures of oxidative stress in bronchial epithelial cell models injured by cigarette smoke extract. Int. J. Mol. Sci..

[12] Koarai A., Sugiura H., Yanagisawa S., Ichikawa T., Minakata Y., Matsunaga K., Hirano T., Akamatsu K., Ichinose M. (2010). Oxidative stress enhances toll-like receptor 3 response to double-stranded RNA in airway epithelial cells. Am. J. Respir. Cell Mol. Biol..

[13] Kayalar Ö., Rajabi H., Konyalilar N., Mortazavi D., Aksoy G.T., Wang J., Bayram H. (2024). Impact of particulate air pollution on airway injury and epithelial plasticity; underlying mechanisms. Front. Immunol..

[14] Piao C.H., Fan Y., Van Nguyen T., Shin H.S., Kim H.T., Song C.H., Chai O.H. (2021). PM2.5 exacerbates oxidative stress and inflammatory response through the Nrf2/NF-κB signaling pathway in OVA-induced allergic rhinitis mouse model. Int. J. Mol. Sci..

[15] Land W.G. (2021). Role of DAMPs in respiratory virus-induced acute respiratory distress syndrome—with a preliminary reference to SARS-CoV-2 pneumonia. Gene Immun..

[16] Burgoyne R.A., Fisher A.J., Borthwick L.A. (2021). The role of epithelial damage in the pulmonary immune response. Cells.

[17] Heijink I.H., Postma D.S., Noordhoek J.A., Broekema M., Kapus A. (2010). House dust mite–promoted epithelial-to-mesenchymal transition in human bronchial epithelium. Am. J. Respir. Cell Mol. Biol..

[18] Grainge C.L., Davies D.E. (2013). Epithelial injury and repair in airways diseases. Chest.

[19] Bammert M.-T., Kollak I., Hoffmann J., Peter E., Ansari M., Schlüter H., Li J., Campos A.R., Viollet C., Gantner F. (2025). Dual roles of fibroblast-epithelial crosstalk in acute and chronic lung injury. J. Biol. Chem..

[20] Lucas C.D., Medina C.B., Bruton F.A., Dorward D.A., Raymond M.H., Tufan T., Etchegaray J.I., Barron B., Oremek M.E.M., Arandjelovic S. (2022). Pannexin 1 drives efficient epithelial repair after tissue injury. Sci. Immunol..

[21] McCauley K.B., Kukreja K., Tovar Walker A.E., Jaffe A.B., Klein A.M. (2024). A map of signaling responses in the human airway epithelium. Cell Syst..

[22] Rock J.R., Onaitis M.W., Rawlins E.L., Lu Y., Clark C.P., Xue Y., Randell S.H., Hogan B.L.M.M. (2009). Basal cells as stem cells of the mouse trachea and human airway epithelium. Proc. Natl. Acad. Sci..

[23] Watanabe S., Alexander M., Misharin A.V., Budinger G.R.S. (2019). The role of macrophages in the resolution of inflammation. J. Clin. Investig..

[24] Krzyszczyk P., Schloss R., Palmer A., Berthiaume F. (2018). The role of macrophages in acute and chronic wound healing and interventions to promote pro-wound healing phenotypes. Front. Physiol..

[25] Bissonnette E.Y., Lauzon-Joset J.-F., Debley J.S., Ziegler S.F. (2020). Cross-Talk between alveolar macrophages and lung epithelial cells is essential to maintain lung homeostasis. Front. Immunol..

[26] Sacco O., Silvestri M., Sabatini F., Sale R., Defilippi A.-C., Rossi G.A. (2004). Epithelial cells and fibroblasts: structural repair and remodelling in the airways. Paediatr. Respir. Rev..

[27] Phogat S., Thiam F., Al Yazeedi S., Abokor F.A., Osei E.T. (2023). 3D in vitro hydrogel models to study the human lung extracellular matrix and fibroblast function. Respir. Res..

[28] Gray T.E., Guzman K., Davis C.W., Abdullah L.H., Nettesheim P. (1996). Mucociliary differentiation of serially passaged normal human tracheobronchial epithelial cells. Am. J. Respir. Cell Mol. Biol..

[29] Castaneda D.C., Jangra S., Yurieva M., Martinek J., Callender M., Coxe M., Choi A., García-Bernalt Diego J., Wu T.-C., Marches F. (2023). Protocol for establishing primary human lung organoid-derived air-liquid interface cultures from cryopreserved human lung tissue. STAR Protoc..

[30] Manna V., Caradonna S. (2021). Isolation, expansion, differentiation, and histological processing of human nasal epithelial cells. STAR Protoc..

[31] Wijesekara P., Yadav P., Perkins L.A., Stolz D.B., Franks J.M., Watkins S.C., Reinoso Jacome E., Brody S.L., Horani A., Xu J. (2022). Engineering rotating apical-out airway organoid for assessing respiratory cilia motility. iScience.

[32] Leach T., Gandhi U., Reeves K.D., Stumpf K., Okuda K., Marini F.C., Walker S.J., Boucher R., Chan J., Cox L.A. (2023). Development of a novel air–liquid interface airway tissue equivalent model for in vitro respiratory modeling studies. Sci. Rep..

[33] Ding P., Wu H., Fang L., Wu M., Liu R. (2014). Transmigration and phagocytosis of macrophages in an airway infection model using four-dimensional techniques. Am. J. Respir. Cell Mol. Biol..

[34] van Riet S., van Schadewijk A., de Vos S., Vandeghinste N., Rottier R.J., Stolk J., Hiemstra P.S., Khedoe P. (2020). Modulation of airway epithelial innate immunity and wound repair by M(GM-CSF) and M(M-CSF) macrophages. J. Innate Immun..

[35] Kortekaas R.K., Geillinger-Kästle K.E., Fuentes-Mateos R., van Orsoy R., Al-Alyan N., Burgess J.K., Gosens R. (2024). The disruptive effects of COPD exacerbation-associated factors on epithelial repair responses. Front. Immunol..

[36] Alber A.B., Marquez H.A., Ma L., Kwong G., Thapa B.R., Villacorta-Martin C., Lindstrom-Vautrin J., Bawa P., Wang F., Luo Y. (2023). Directed differentiation of mouse pluripotent stem cells into functional lung-specific mesenchyme. Nat. Commun..

[37] Albers S., Thiebes A.L., Gessenich K.L., Jockenhoevel S., Cornelissen C.G. (2016). Differentiation of respiratory epithelium in a 3-dimensional co-culture with fibroblasts embedded in fibrin gel. Multidiscip. Respir. Med..

[38] Temann A., Golovina T., Neuhaus V., Thompson C., Chichester J.A., Braun A., Yusibov V. (2017). Evaluation of inflammatory and immune responses in long-term cultured human precision-cut lung slices. Hum. Vaccines Immunother..

[39] Neuhaus V., Schaudien D., Golovina T., Temann U.-A., Thompson C., Lippmann T., Bersch C., Pfennig O., Jonigk D., Braubach P. (2017). Assessment of long-term cultivated human precision-cut lung slices as an ex vivo system for evaluation of chronic cytotoxicity and functionality. J. Occup. Med. Toxicol..

[40] Henjakovic M., Sewald K., Switalla S., Kaiser D., Müller M., Veres T.Z., Martin C., Uhlig S., Krug N., Braun A. (2008). Ex vivo testing of immune responses in precision-cut lung slices. Toxicol. Appl. Pharmacol..

[41] Switalla S., Lauenstein L., Prenzler F., Knothe S., Förster C., Fieguth H.G., Pfennig O., Schaumann F., Martin C., Guzman C.A. (2010). Natural innate cytokine response to immunomodulators and adjuvants in human precision-cut lung slices. Toxicol. Appl. Pharmacol..

[42] Akram K.M., Yates L.L., Mongey R., Rothery S., Gaboriau D.C.A., Sanderson J., Hind M., Griffiths M., Dean C.H. (2019). Live imaging of alveologenesis in precision-cut lung slices reveals dynamic epithelial cell behaviour. Nat. Commun..

[43] Brown S.M., Koarai A., Sturton R.G., Nicholson A.G., Barnes P.J., Donnelly L.E. (2013). A role for M2 and M3 muscarinic receptors in the contraction of rat and human small airways. Eur. J. Pharmacol..

[44] Schlepütz M., Rieg A.D., Seehase S., Spillner J., Perez-Bouza A., Braunschweig T., Schroeder T., Bernau M., Lambermont V., Schlumbohm C. (2012). Neurally mediated airway constriction in human and other species: a comparative study using precision-cut lung slices (PCLS). PLoS One.

[45] Preuß E.B., Schubert S., Werlein C., Stark H., Braubach P., Höfer A., Plucinski E.K.J., Shah H.R., Geffers R., Sewald K. (2022). The challenge of long-term cultivation of human precision-cut lung slices. Am. J. Pathol..

[46] Bankole E., Wong C.W., Kim S., Hind M., Dean C.H. (2025). A human PCLS model of lung injury and repair for discovery and pharmaceutical research. Respir. Res..

[47] DeTemple D.E., Cammann S., Bahlmann J., Buettner M., Heisterkamp A., Vondran F.W.R., Kalies S.K. (2020). Longitudinal imaging and femtosecond laser manipulation of the liver: how to generate and trace single-cell-resolved micro-damage in vivo. PLoS One.

[48] Donath S., Seidler A.E., Mundin K., Wenzel J., Scholz J., Gentemann L., Kalies J., Faix J., Ngezahayo A., Bleich A. (2023). Epithelial restitution in 3D - revealing biomechanical and physiochemical dynamics in intestinal organoids via fs laser nanosurgery. iScience.

[49] Müller D., Klamt T., Gentemann L., Heisterkamp A., Kalies S.M.K. (2021). Evaluation of laser induced sarcomere micro-damage: role of damage extent and location in cardiomyocytes. PLoS One.

[50] Müller D., Hagenah D., Biswanath S., Coffee M., Kampmann A., Zweigerdt R., Heisterkamp A., Kalies S.M.K. (2019). Femtosecond laser-based nanosurgery reveals the endogenous regeneration of single Z-discs including physiological consequences for cardiomyocytes. Sci. Rep..

[51] Donath S., Angerstein L., Gentemann L., Müller D., Seidler A.E., Jesinghaus C., Bleich A., Heisterkamp A., Buettner M., Kalies S. (2022). Investigation of colonic regeneration via precise damage application using femtosecond laser-based nanosurgery. Cells.

[52] Gentemann L., Donath S., Seidler A.E., Patyk L., Buettner M., Heisterkamp A., Kalies S. (2023). Mimicking acute airway tissue damage using femtosecond laser nanosurgery in airway organoids. Front. Cell Dev. Biol..

[53] König K., Riemann I., Fischer P., Halbhuber K.J. (1999). Intracellular nanosurgery with near infrared femtosecond laser pulses. Cell. Mol. Biol. (Noisy-le-grand)..

[54] Zhou J., Alvarez-Elizondo M.B., Botvinick E., George S.C. (2012). Local small airway epithelial injury induces global smooth muscle contraction and airway constriction. J. Appl. Physiol..

[55] Heisterkamp A., Maxwell I.Z., Mazur E., Underwood J.M., Nickerson J.A., Kumar S., Ingber D.E. (2005). Pulse energy dependence of subcellular dissection by femtosecond laser pulses. Opt. Express.

[56] Lyons-Cohen M.R., Thomas S.Y., Cook D.N., Nakano H. (2017). Precision-cut mouse lung slices to visualize live pulmonary dendritic cells. JoVE J..

[57] Vogel A., Noack J., Hüttman G., Paltauf G. (2005). Mechanisms of femtosecond laser nanosurgery of cells and tissues. Appl. Phys. B.

[58] Burgstaller G., Vierkotten S., Lindner M., Königshoff M., Eickelberg O. (2015). Multidimensional immunolabeling and 4D time-lapse imaging of vital ex vivo lung tissue. Am. J. Physiol. Lung Cell. Mol. Physiol..

[59] Perez J.F., Sanderson M.J. (2005). The frequency of calcium oscillations induced by 5-HT, ACH, and KCl determine the contraction of smooth muscle cells of intrapulmonary bronchioles. J. Gen. Physiol..

[60] Bergner A., Sanderson M.J. (2002). Acetylcholine-induced calcium signaling and contraction of airway smooth muscle cells in lung slices. J. Gen. Physiol..

[61] Neuhaus V., Danov O., Konzok S., Obernolte H., Dehmel S., Braubach P., Jonigk D., Fieguth H.G., Zardo P., Warnecke G. (2018). Assessment of the cytotoxic and immunomodulatory effects of substances in human precision-cut lung slices. JoVE J..

[62] Stringer C., Wang T., Michaelos M., Pachitariu M. (2021). Cellpose: a generalist algorithm for cellular segmentation. Nat. Methods.

[63] Pachitariu M., Stringer C. (2022). Cellpose 2.0: how to train your own model. Nat. Methods.

[64] Schindelin J., Arganda-Carreras I., Frise E., Kaynig V., Longair M., Pietzsch T., Preibisch S., Rueden C., Saalfeld S., Schmid B. (2012). Fiji: an open-source platform for biological-image analysis. Nat. Methods.

[65] Ershov D., Phan M.S., Pylvänäinen J.W., Rigaud S.U., Le Blanc L., Charles-Orszag A., Conway J.R.W., Laine R.F., Roy N.H., Bonazzi D. (2022). TrackMate 7: integrating state-of-the-art segmentation algorithms into tracking pipelines. Nat. Methods.

[66] Button B., Boucher R.C. (2008). Role of mechanical stress in regulating airway surface hydration and mucus clearance rates. Respir. Physiol. Neurobiol..

[67] Adler K.B., Tuvim M.J., Dickey B.F. (2013). Regulated Mucin secretion from airway epithelial cells. Front. Endocrinol..

[68] Graf J., Trautmann-Rodriguez M., Sabnis S., Kloxin A.M., Fromen C.A. (2023). On the path to predicting immune responses in the lung: modeling the pulmonary innate immune system at the air-liquid interface (ALI). Eur. J. Pharmaceut. Sci..

[69] Davis C.W., Dowell M.L., Lethem M., Van Scott M. (1992). Goblet cell degranulation in isolated canine tracheal epithelium: response to exogenous ATP, ADP, and adenosine. Am. J. Physiol. Cell Physiol..

[70] Abohalaka R. (2023). Bronchial epithelial and airway smooth muscle cell interactions in health and disease. Heliyon.

[71] Zhou J., Alvarez-Elizondo M.B., Botvinick E., George S.C. (2013). Adenosine A1 and prostaglandin E receptor 3 receptors mediate global airway contraction after local epithelial injury. Am. J. Respir. Cell Mol. Biol..

[72] Kemp P.A., Sugar R.A., Jackson A.D. (2004). Nucleotide-mediated mucin secretion from differentiated human bronchial epithelial cells. Am. J. Respir. Cell Mol. Biol..

[73] Shishikura Y., Koarai A., Aizawa H., Yamaya M., Sugiura H., Watanabe M., Hashimoto Y., Numakura T., Makiguti T., Abe K. (2016). Extracellular ATP is involved in dsRNA-induced MUC5AC production via P2Y2R in human airway epithelium. Respir. Res..

[74] Kim K.C., Lee B.C. (1991). P 2 purinoceptor regulation of mucin release by airway goblet cells in primary culture. Br. J. Pharmacol..

[75] Button B., Okada S.F., Frederick C.B., Thelin W.R., Boucher R.C. (2013). Mechanosensitive ATP release maintains proper mucus hydration of airways. Sci. Signal..

[76] White S.R. (2011). Apoptosis and the airway epithelium. J. Allergy.

[77] Tadokoro T., Gao X., Hong C.C., Hotten D., Hogan B.L.M. (2016). BMP signaling and cellular dynamics during regeneration of airway epithelium from basal progenitors. Development.

[78] Erjefält J.S., Erjefält I., Sundler F., Persson C.G.A. (1995). In vivo restitution of airway epithelium. Cell Tissue Res..

[79] Puchelle E., Zahm J.-M., Tournier J.-M., Coraux C. (2006). Airway epithelial repair, regeneration, and remodeling after injury in chronic obstructive pulmonary disease. Proc. Am. Thorac. Soc..

[80] Xie B., Laxman B., Hashemifar S., Stern R., Gilliam T.C., Maltsev N., White S.R. (2018). Chemokine expression in the early response to injury in human airway epithelial cells. PLoS One.

[81] Gindele J.A., Mang S., Pairet N., Christ I., Gantner F., Schymeinsky J., Lamb D.J. (2017). Opposing effects of in vitro differentiated macrophages sub-type on epithelial wound healing. PLoS One.

[82] Kretschmer S., Pieper M., Klinger A., Hüttmann G., König P. (2017). Imaging of wound closure of small epithelial lesions in the mouse trachea. Am. J. Pathol..

[83] Kupfer A., Dennert G., Singer S.J. (1983). Polarization of the Golgi apparatus and the microtubule-organizing center within cloned natural killer cells bound to their targets. Proc. Natl. Acad. Sci..

[84] Nobes C.D., Hall A. (1999). Rho GTPases control polarity, protrusion, and adhesion during cell movement. J. Cell Biol..

[85] Zahm J.-M., Kaplan H., Hérard A.-L., Doriot F., Pierrot D., Somelette P., Puchelle E. (1997). Cell migration and proliferation during the in vitro wound repair of the respiratory epithelium. Cell Motil Cytoskeleton.

[86] Hoffmann W. (2005). TFF (trefoil factor family) peptide-triggered signals promoting mucosal restitution. Preprint.

[87] Heijink I.H., Kuchibhotla V.N.S., Roffel M.P., Maes T., Knight D.A., Sayers I., Nawijn M.C. (2020). Epithelial cell dysfunction, a major driver of asthma development. Allergy.

[88] Russell R.J., Boulet L.-P., Brightling C.E., Pavord I.D., Porsbjerg C., Dorscheid D., Sverrild A. (2024). The airway epithelium: an orchestrator of inflammation, a key structural barrier and a therapeutic target in severe asthma. Eur. Respir. J..

[89] Spann K., Snape N., Baturcam E., Fantino E. (2016). The impact of early-life exposure to air-borne environmental insults on the function of the airway epithelium in asthma. Ann. Glob. Health.

[90] Kageyama T., Ito T., Tanaka S., Nakajima H. (2024). Physiological and immunological barriers in the lung. Semin. Immunopathol..

[91] Engler A.E., Ysasi A.B., Pihl R.M.F., Villacorta-Martin C., Heston H.M., Richardson H.M.K., Thapa B.R., Moniz N.R., Belkina A.C., Mazzilli S.A. (2020). Airway-associated macrophages in homeostasis and repair. Cell Rep..

[92] Ysasi A.B., Engler A.E., Bawa P.S., Wang F., Conrad R.D., Yeung A.K., Rock J.R., Beane-Ebel J., Mazzilli S.A., Franklin R.A. (2024). A specialized population of monocyte-derived tracheal macrophages promote airway epithelial regeneration through a CCR2-dependent mechanism. iScience.

[93] Chang S.-Y., Chang W.-H., Yang D.C., Hong Q.-S., Hsu S.-W., Wu R., Chen C.-H. (2025). Autologous precision-cut lung slice co-culture models for studying macrophage-driven fibrosis. Front. Physiol..

[94] Wang J., Hossain M., Thanabalasuriar A., Gunzer M., Meininger C., Kubes P. (2017). Visualizing the function and fate of neutrophils in sterile injury and repair. Science.

[95] Zemans R.L., Briones N., Campbell M., McClendon J., Young S.K., Suzuki T., Yang I.V., De Langhe S., Reynolds S.D., Mason R.J. (2011). Neutrophil transmigration triggers repair of the lung epithelium via β-catenin signaling. Proc. Natl. Acad. Sci..

[96] Kooistra T., Saez B., Roche M., Egea-Zorrilla A., Li D., Anketell D., Nguyen N., Villoria J., Gillis J., Petri E. (2025). Airway basal stem cells are necessary for the maintenance of functional intraepithelial airway macrophages. Cell Rep..

[97] Hoffmann F.M., Berger J.L., Lingel I., Laumonnier Y., Lewkowich I.P., Schmudde I., König P. (2018). Distribution and interaction of Murine pulmonary phagocytes in the naive and allergic lung. Front. Immunol..

[98] Puttur F., Gregory L.G., Lloyd C.M. (2019). Airway macrophages as the guardians of tissue repair in the lung. Immunol. Cell Biol..

[99] Adams W., Espicha T., Estipona J. (2021). Getting your neutrophil: neutrophil transepithelial migration in the lung. Infect. Immun..

[100] Deng Y., Herbert J.A., Smith C.M., Smyth R.L. (2018). An in vitro transepithelial migration assay to evaluate the role of neutrophils in Respiratory Syncytial Virus (RSV) induced epithelial damage. Sci. Rep..

[101] Kreisel D., Nava R.G., Li W., Zinselmeyer B.H., Wang B., Lai J., Pless R., Gelman A.E., Krupnick A.S., Miller M.J. (2010). In vivo two-photon imaging reveals monocyte-dependent neutrophil extravasation during pulmonary inflammation. Proc. Natl. Acad. Sci..

[102] Bordoni V., Matusali G., Mariotti D., Antonioli M., Cimini E., Sacchi A., Tartaglia E., Casetti R., Grassi G., Notari S. (2022). The interplay between SARS-CoV-2 infected airway epithelium and immune cells modulates regulatory/inflammatory signals. iScience.

[103] Chen S.M., Cheng D.-S., Williams B.J., Sherrill T.P., Han W., Chont M., Saint-Jean L., Christman J.W., Sadikot R.T., Yull F.E. (2008). The nuclear factor kappa-B pathway in airway epithelium regulates neutrophil recruitment and host defence following *Pseudomonas aeruginosa* infection. Clin. Exp. Immunol..

[104] Basil M.C., Morrisey E.E. (2020). Lung regeneration: a tale of mice and men. Semin. Cell Dev. Biol..

[105] Matute-Bello G., Frevert C.W., Martin T.R. (2008). Animal models of acute lung injury. Am. J. Physiol. Lung Cell. Mol. Physiol..

[106] Russell W.M.S. (2005). The three Rs: past, present and future. Anim. Welf..

