# Supplementary material for: Live imaging of human airway epithelial repair in precision-cut lung slices after targeted cell damage
Source: Biochem Biophys Rep. 2026 Jan 29;45:102470. doi: 10.1016/j.bbrep.2026.102470 (PMC12876308; doi:10.1016/j.bbrep.2026.102470)
Supplement: Supplementary Material [file mmc1.pdf]

Supplemental Material

Live Imaging of Human Airway Epithelial Repair in Precision-Cut Lung Slices after Targeted Cell Damage

Lara Gentemann, Fabian Röpken, Philipp Joel Mroch, Nils Noltemeyer, Sören Donath, Anna E. Seidler, Christopher Werlein, Patrick Zardo, Lavinia Neubert, Danny Jonigk, Hans-Gerd Fieguth, Alexander Heisterkamp, Katherina Sewald, Stefan Kalies**

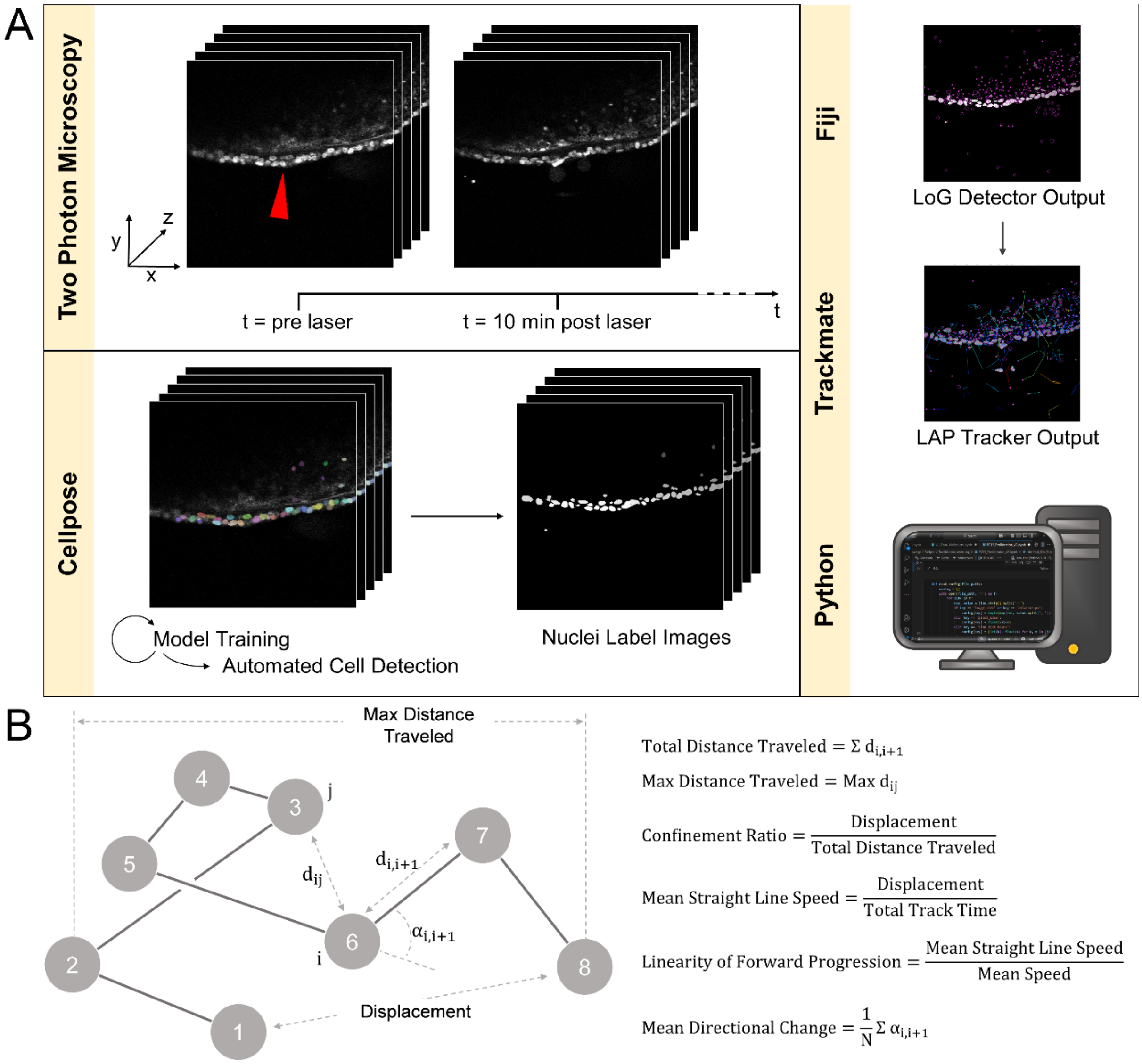

Figure S1: Schematic illustration of cell movement image analysis pipeline. (A): Two-photon microscopy image stacks of hPCLS airways stained with Hoechst were captured from pre to up to 12 hours post laser-based cell ablation (top left). For automated cell nuclei detection, Cellpose was employed. Upon training a custom model, nuclei label images were generated (bottom left). Further analysis was carried out via Fiji and Trackmate plugin and/or final computation of data using custom Python scripts for determination of cell counts and cell track features (right). (B): Definitions of cell track features.

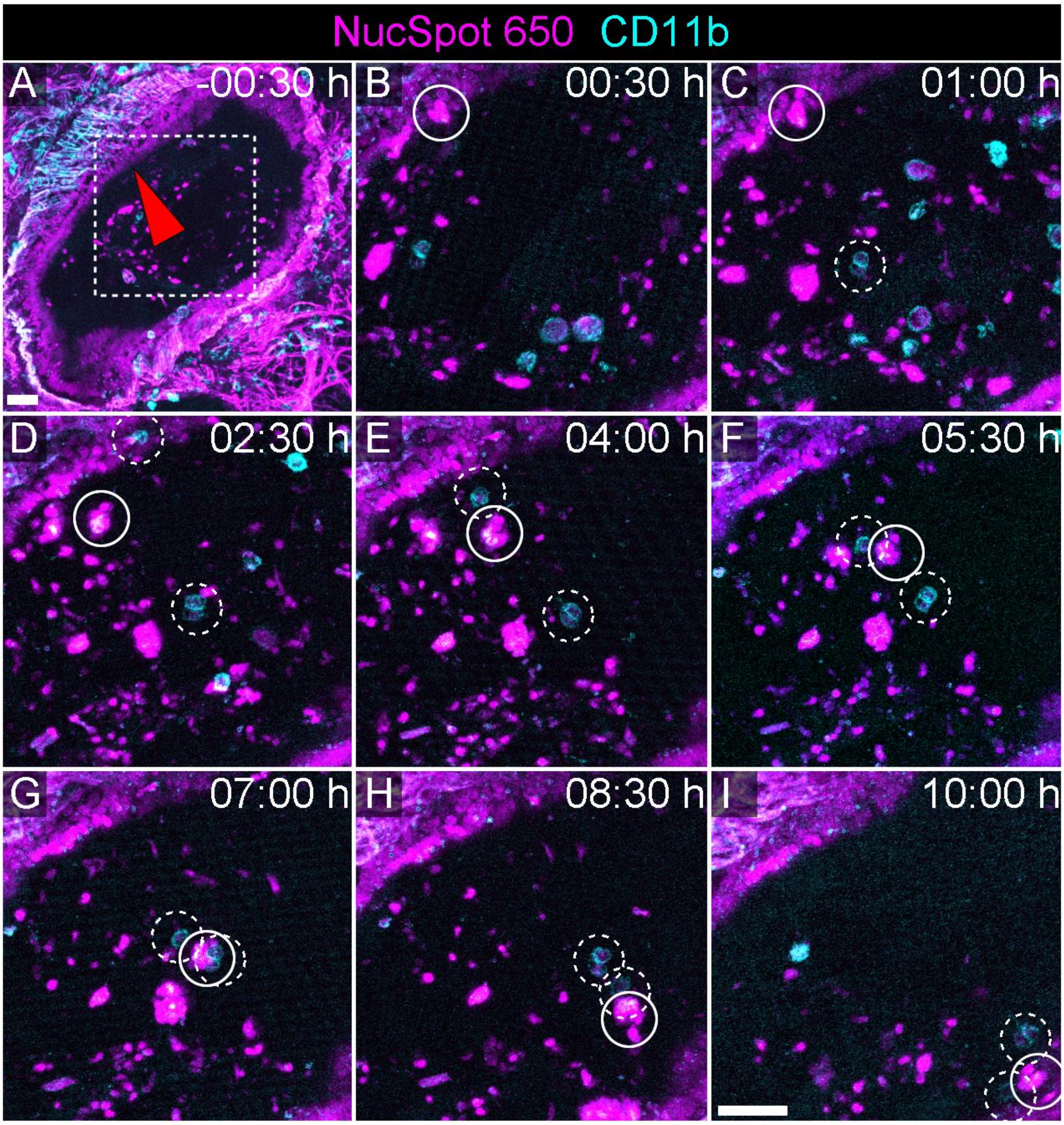

Figure S2: Attraction of CD11b-positive cells by laser-induced damage site/cell debris. (A-I): Time series of confocal microscopy images showing a hPCLS airway stained with NucSpot® Live 650 (magenta) and PE-labeled anti-CD11b (cyan) (max. intensity z-projection of captures ±50 µm of ablation site) at pre (A), 0.5 h (B), 1 h (C), 2.5 h (D), 4 h (E), 5.5 h (F), 7 h (G), 8.5 h (H) and 10 h (I) post laser-based damage induction, displaying cell debris being shed into the lumen (closed circle) and attracting CD11b-positive cells (dotted circles). (A) shows the entirely captured airway with site of ablation indicated by red arrow, (B-I) show the region framed by the dotted box in (A). Presented data was observed in n=1 experiment. Scale bars: 50 µm.

Video S1: hPCLS mucus layer restitutes upon targeted cell ablation, related to Figure 2.

Representative time series of wheat germ agglutinin (WGA)-Alexa Fluor 488-stained hPCLS confocal microscopy images (max. intensity z-projection of captures ±65 µm of ablation site) from pre to 24 h

post laser-based ablation of epithelial cells, corresponding to data shown in Figure 2. Red arrow: site of ablation; WGA: mucus stain. Scale bar: 50 µm.

Video S2: hPCLS epithelial repair involves cellular movement, related to Figure 3.

Representative time series of NucSpot® Live 650-stained hPCLS confocal microscopy images (max. intensity z-projection of captures ±25 µm of ablation site) from pre to 24 h post laser-based ablation of epithelial cells, corresponding to data shown in Figure 3. Red arrow: site of cell ablation, NucSpot® Live 650: nuclear stain. Scale bar: 50 µm.

Video S3: Recruitment of presumably immune cells to damage site, related to Figure 5.

Exemplary time series of Hoechst- (red) and Calcein-AM- (green) stained hPCLS multiphoton microscopy images (max. intensity z-projection of captures ±25 µm of ablation site) from pre to 7:10 h post laser-based ablation of epithelial cells, corresponding to data shown in Figure 5. Scale bar: 50 µm.

Video S4: Representative control airway.

Exemplary time series of Hoechst- (red) and Calcein-AM- (green) stained hPCLS multiphoton microscopy images (max. intensity z-projection of captures ±25 µm of ablation site) within the time course of 8 h. Scale bar: 50 µm.

Video S5: Attraction of CD11b-positive cells by laser-induced damage site/cell debris, related to Figure S2.

Time series of confocal microscopy images showing a hPCLS airway stained with NucSpot® Live 650 (magenta) and PE-labeled anti-CD11b (cyan) (max. intensity z-projection of captures ±50 µm of ablation site) from pre to 10 h post laser-based damage induction, displaying cell debris being shed into the lumen (closed circle) and attracting CD11b-positive cells (dotted circles)., corresponding to data shown in Figure S2. Scale bars: 50 µm.
